# Clinicopathological Characteristics and Short-Term Survival Analyses of Cutaneous Malignant Melanoma

**DOI:** 10.7759/cureus.9868

**Published:** 2020-08-19

**Authors:** Faizan Ullah, Muhammad Jamshed, Osama Shakeel, Uzma Khalid, Romaisa Khan, Rahim Dhanani, Rida Mansoor, Sami Ullah, Raza Hussain

**Affiliations:** 1 Surgical Oncology, Shaukat Khanum Memorial Cancer Hospital and Research Centre, Lahore, PAK; 2 Internal Medicine, Shrewsbury and Telford Hospital NHS Trust, Shrewsbury, GBR; 3 Surgical Oncology, Shaukat Khanum Memoiral Cancer Hospital and Research Centre, Lahore, PAK; 4 Internal Medicine, Jinnah Postgraduate Medical Centre, Karachi, PAK; 5 Plastic and Reconstructive Surgery, Shaukat Khanum Memoiral Cancer Hospital and Research Centre, Lahore, PAK

**Keywords:** malignant melanoma, cutaneous malignant melanoma, non mucosal malignant melanoma, survival analyses, pakistan

## Abstract

Objective

Cutaneous malignant melanoma (CMM) arises from melanocytes, which are pigment-producing cells in the skin. CMM constitutes less than 5% of all cutaneous malignancies worldwide but is associated with the highest mortality rate among all skin cancers. The objective of this study was to examine the profile of clinicopathological factors, survival analyses, recurrence rate, metastatic rates, and the management of CMM at Shaukat Khanum Memorial Cancer Hospital and Research Centre (SKMCH&RC) in Lahore, Pakistan.

Methodology

All patients with a diagnosis of CMM treated at our institute from 2014 to 2017 were included in the study. Demographic variables and clinicopathological characteristics were collected and short-term oncological outcomes were recorded. All data were entered and analyzed in SPSS Statistics version 21 (IBM, Armonk, NY).

Results

A total of 28 patients were included in the study. The median age of the patients was 46.5 ±15.9 years. There were 16 male and 12 female patients. A family history of melanoma was present in 7.1% (n=2) of the patients. All patients had a mean survival of 13.43 ±9.09 months. The lower limb was the most common site of tumor among all patients, accounting for 46.4% (n=13) of the cases. On histopathological analyses, ulceration was seen in 53.6% (n=15) of the patients. Unclassified tumor type was present in 75% (n=21) of the patients, followed by nodular in 21.4% (n=6), and superficial spreading in 3.5% (n=1). Clark level IV was the most common presentation, constituting 46.4% (n=13) of the cases. Metastasis was seen in 50% (n=14) of the patients. Local recurrence was observed in 60.7% (n=17) of the patients; 64.3% (n=18) of the patients were alive after one year of treatment.

Conclusion

CMM is a disease with very high fatality rates. Although it is a disease commonly associated with fair-skinned populations, the incidence of CMM is rising in our part of the world as well. Early diagnosis and prompt management of the disease are crucial in its treatment. However, the mortality rate associated with this disease is still not favorable.

## Introduction

Cutaneous malignant melanoma (CMM) arises from melanocytes, which are pigment-producing cells in the skin. Even though CMM accounts for less than 5% of all cutaneous malignancies worldwide, it is associated with the highest mortality rates among all skin cancers [1]. As per 2008 estimates, 200,000 new cases of CMM were recorded globally, and 46,000 people died from the disease [2]. CMM is responsible for up to 80% of all deaths due to malignant melanoma [3].

CMM is a disease commonly associated with fair-skinned populations, and hence Scandinavian countries have the highest incidence rates of CMM in the world [4]. The overall incidence of CMM has increased over the last few decades [5]. The rationale behind this trend is the rise of sun-seeking behavior among people in many parts of the world [6]. CMM is a multifactorial disease in which environmental, genetic, and individual host factors play their roles, as evident from immigrant studies [7]. Common risk factors for CMM are fair skin and hair, presence of atypical nevi, light eyes, advanced age, male gender, and increased exposure to ultraviolet (UV) radiation [8,9].

CMM is of high concern because it is associated with high morbidity and mortality rates [10]. As per the American Joint Commission for Cancer (AJCC), five-year survival for early-stage CMM ranges from 98% for localized melanoma to 68% for regional disease (clinical stage III) [11]. Metastatic melanoma is a fatal disease with a five-year survival rate of 17% [11]. However, survival outcomes are improving with the emergence of better treatment options. In a majority of the cases, patients with CMM present for treatment in the early stages; however, patients tend to develop metastases through the course of the treatment [11]. The most common metastatic sites are the lungs, liver, bone, and brain [12]. According to the data in the published literature, one-third of CMM patients will develop recurrence [13], and most of the patients experience metastatic involvement within three years [12,14-17]. Hence, patients should be kept under thorough and strict surveillance for at least two years following the diagnosis [14].

Unfortunately, there is a paucity in the literature regarding the studies addressing the clinicopathological factors and survival analyses relating to CMM from South Asia. This is due to unreliable centralized cancer-registries, the absence of compulsory notification, and the lack of health-seeking behavior among the general public in this region. The present study intends to describe the profile of the clinicopathological factors, survival analyses, recurrence rate, metastatic rates, and the management of the disease. As there is a scarcity of data related to CMM from Pakistan, we hope this study will fill the gaps and will help to plan strategies for the prompt diagnosis and treatment of cutaneous melanoma, in order to decrease the morbidity and mortality associated with it.

## Materials and methods

We performed a cross-sectional retrospective review of all patients who were registered at Shaukat Khanum Memorial Cancer Hospital and Research Centre (SKMCH&RC) with a diagnosis of malignant melanoma. From this data, we identified those patients who had CMM, or cutaneous melanoma. SKMCH&RC is a dedicated cancer hospital that accepts patients from all over Pakistan, with the bulk of the patients hailing from the northern half of the country. The hospital also accepts patients from the bordering areas of Afghanistan.

All patients with a diagnosis of CMM treated at our institute from 2014 to 2017 were included in the study. Ethical approval was sought from the Institutional Review Board (IRB) of SKMCH&RC. Informed consent was obtained prior to the collection of data. Patients with a second malignancy were excluded from the study. Data were collected through the human information system (electronic data) at SKMCH&RC.

Calculations were performed with SPSS Statistics version 21 (IBM, Armonk, NY). Data were presented using medians with minimum and maximum value for skewly distributed quantitative variables. For categorical variables, frequency and percentages were recorded. For survival analyses, the Kaplan-Meier curve was applied.

## Results

A total of 28 patients were included in the study. The mean age of the patients was 46.5 ±15.9 years. Most of the patients were between 41 to 60 years of age (53.6%, n=15). Most of the patients were males (57.1%, n=16) while females constituted 42.9% (n=12) of the patient population;. 50% (n=14) of the patients were from Khyber Pakhtunkhwa (northern areas of Pakistan), followed by Punjab (39.3%, n=11), and Afghanistan (10.7%, n=3). Two patients had other malignancies. Five patients had a history of addiction, three patients smoked cigarettes, and two chewed tobacco. A family history of melanoma was present in 7.1% (n=2) of the patients. The lower limb was the most common site of tumor among all patients, accounting for 46.4% (n=13) of the cases.

On histopathological analyses, ulceration was seen in 53.6% (n=15) of the patients. Unclassified tumor type was present in 75% (n=21) of the patients, followed by nodular in 21.4% (n=6), and superficial spreading in 3.5% (n=1). Clark level IV was the most common presentation, constituting 46.4% (n=13) of the cases. Lymphovascular invasion was seen in 10.7% (n=3) of the patient population. Pathological T4 was the most commonly encountered staging (61.9%, n=13) among the patients. Margins were involved in 18.5% (n=5) of the patients. Metastasis was seen in 50% (n=14) of the patients. The most common site of metastases was lungs (42.9%, n=6). CT scan was the most frequently used modality to detect metastases (50%, n=7) in the patients. Palliative radiotherapy was the most frequently offered treatment method (35.7%, n=5) among the patients with metastases.

**Figure 1:**
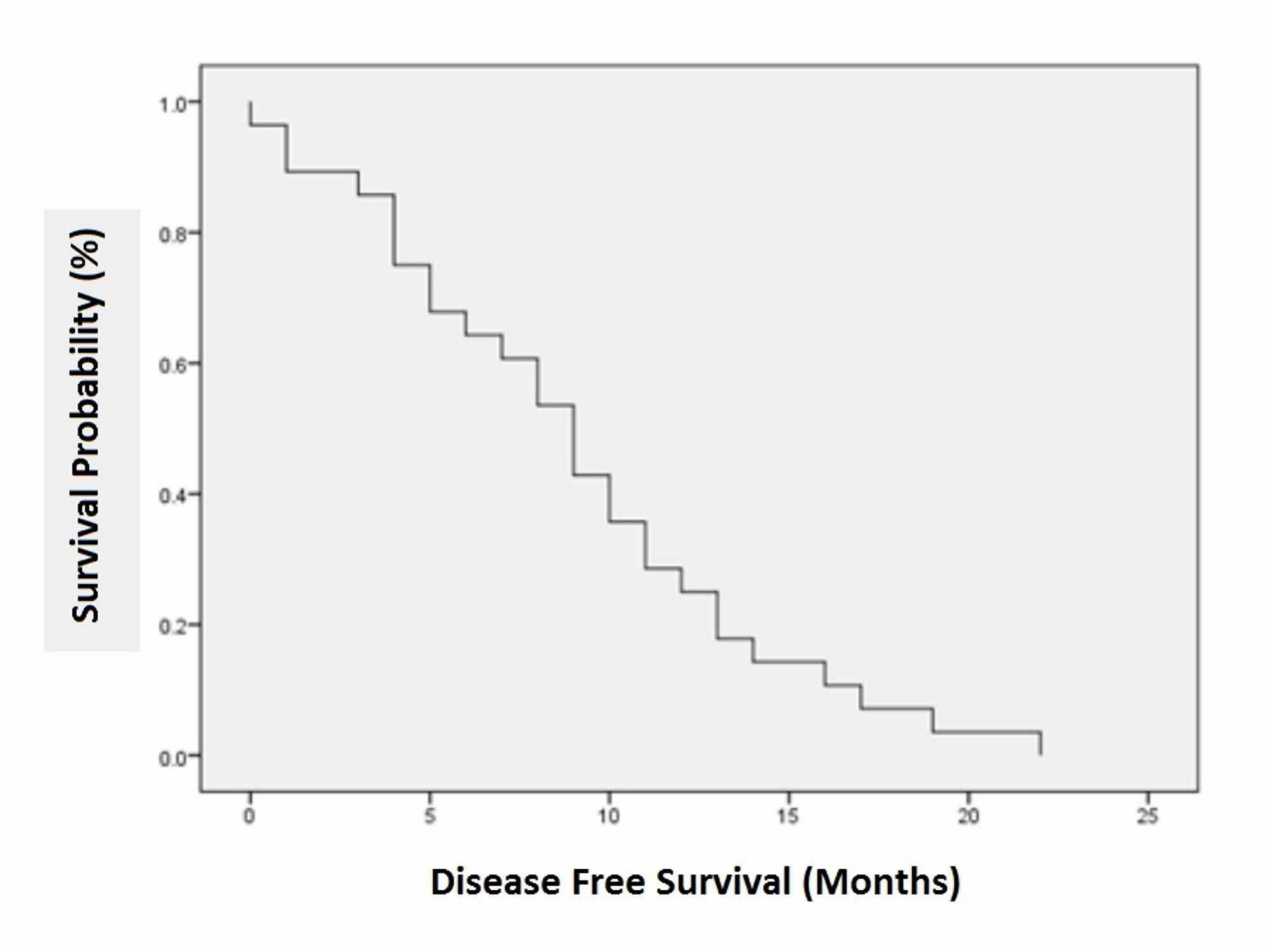
Disease-free survival (in months) among cutaneous malignant melanoma patients at Shaukat Khanum Memorial Cancer Hospital and Research Centre

**Figure 2:**
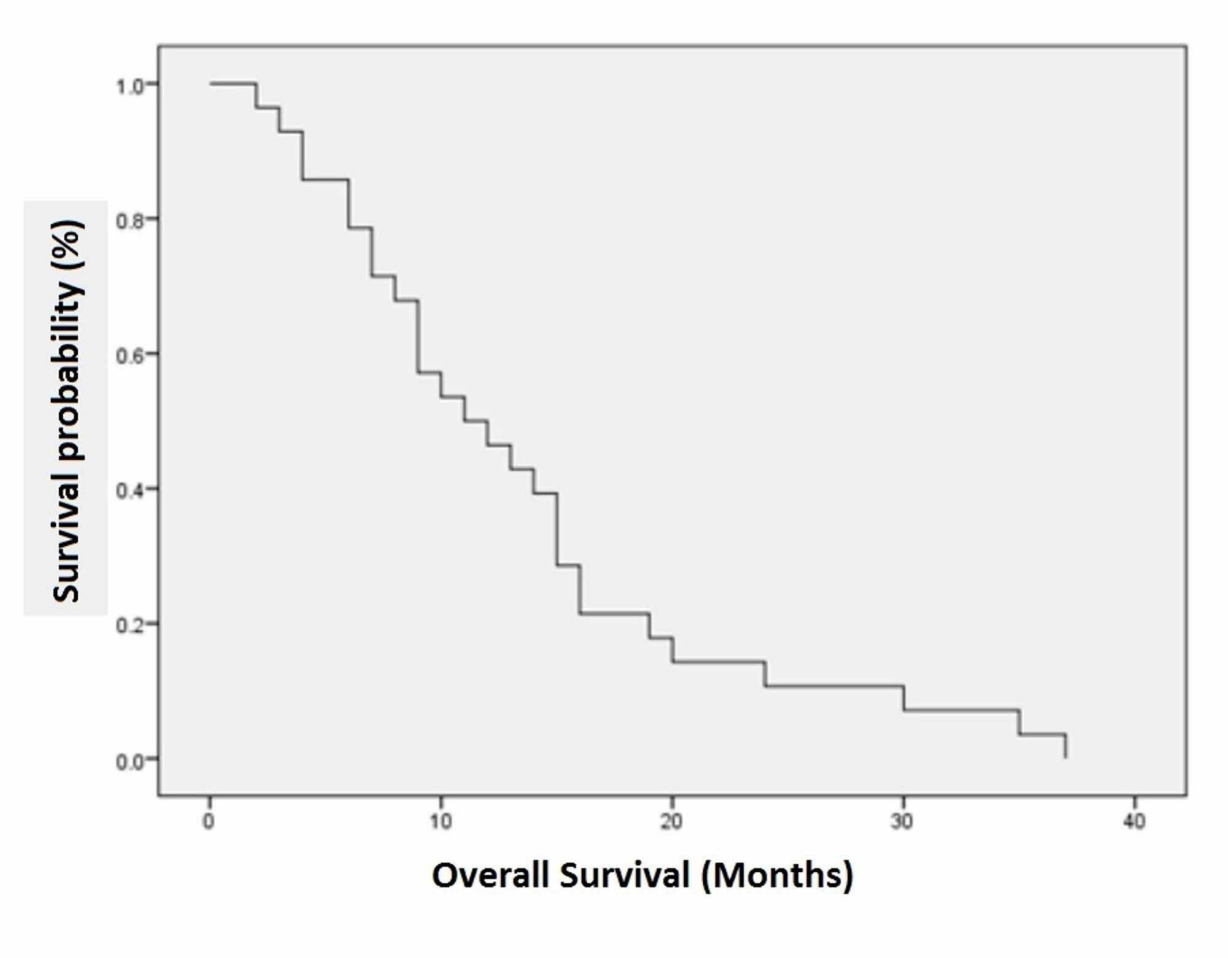
Overall survival (in months) among cutaneous malignant melanoma patients at Shaukat Khanum Memorial Cancer Hospital and Research Centre

Local recurrence was observed in 60.7% (n=17) of the patients. Regional lymph node involvement was the most common site, constituting 88.2% (n=15) of the cases, followed by recurrent mass in 11.8% (n=2). Surgery (wide local excision) was the preferred treatment for loco-regional recurrence and was performed in 41.2% (n=7) of the patients; 64.3% (n=18) of the patients were alive after one year of treatment. The mean disease-free survival was 8.96 ±5.52 months. The mean overall survival was 13.43 ±9.09 months (Tables 1, 2; Figures 1, 2).

**Table 1:** Clinicopathological characteristics of cutaneous malignant melanoma patients

Variables	Frequency	Percentage
Age group (years)		
0-20	3	10.7
21-40	6	17.9
41-60	15	53.6
61-80	4	14.3
81-100	1	3.6
Location of tumor		
Lower limb	13	46.4
Head and neck	6	21.4
Upper limb	6	21.4
Trunk	3	10.7
Clark level		
I	1	3.6
II	1	3.6
III	1	3.6
IV	13	46.4
V	5	17.9
Missing	7	25
Pathological T stage		
T1	1	3.6
T2	3	10.7
T3	4	14.3
T4	13	46.4
Missing	7	25

**Table 2:** Management of recurrence and survival outcomes CT: computed tomography; PET: positron emission tomography; MRI: magnetic resonance imaging

Variables	Frequency	Percentage
Modality to detect metastases		
CT scan	7	50
PET scan	4	28.6
MRI scan	3	21.4
Site of metastases		
Lungs	6	42.9
Bones	5	35.7
Brain	1	7.1
Lymph nodes	1	7.1
Liver	1	7.1
Treatment of metastases		
Palliative radiotherapy	5	35.7
Chemotherapy	3	21.4
Palliation	3	21.4
Radiotherapy	2	14.3
Surgery	1	7.1
Treatment after recurrence		
Surgery alone	7	41.2
Palliative radiotherapy	4	23.5
Surgery + radiotherapy	3	17.6
Chemotherapy	2	11.8
Radiotherapy	1	5.9
Status		
Alive	18	64.3
Dead	6	21.4
Lost to follow-up	4	14.3

## Discussion

Malignant melanoma is the skin cancer arising from pigmented cells of the skin called melanocytes. Even though it is a rare entity compared to other skin cancers, it is associated with a high mortality rate. This tumor can arise either from primary malignant changes within the melanocytes or from pre-existing lesions, which have a tendency to develop malignancy under a favorable environment, for example, dysplastic nevus [18].

We studied the patients who presented to SKMCH&RC with CMM to highlight the important clinicopathological factors responsible for the disease in our population. Our study involved 28 patients presenting to the institution from 2014 to 2017. Our objective was to understand the disease patterns; however, this data is not enough to elucidate a definitive management plan, but this will allow us to add the necessary information to fill in the missing pieces in the existing data while compiling the worldwide data for the better care of patients.

Due to the small sample size and the absence of any significant differences between reported cases of both genders, it is difficult to form an opinion as to whether CMM is more common among males or females in our population. CMM is rare in the younger population and our study confirmed this trend. While previous studies have reported a high incidence among adults of 20-40 years of age, we found a higher incidence among people of 41-60 years of age [18].

Our findings also endorse the theory that patients with a positive family history are at a higher risk of developing malignant melanoma. As far as the site of the tumor is concerned, the anatomical site of CMM tends to vary depending upon factors such as cultural diversity, lifestyle, and societal factors, which affect the mode of clothing and outdoor leisure activities among people [19]. We found a high incidence in lower limbs, followed by head and neck, and upper limbs among our patients, while a very low incidence of trunk lesions was found. This trend can be the result of the clothing culture of Pakistani people, which exposes the lower part of legs to the sun at a significantly higher rate compared to the head, neck, upper limbs, and thorax. The incidence of CMM increases with increased exposure to the UV rays of the sun [20].

T4 stage was the most common presentation in our setup, which could be attributed to the lack of education and awareness about the disease. People are generally unaware as to when they should get the biopsy done, which leads to late presentations of the disease. We found lymphovascular invasion in 10.7% of the patients on histopathology due to similar reasons. Most of the patients presented with Clark IV disease. More than half of the patients presented with ulceration. The nodal spread was more common when compared to superficial spread. The locoregional recurrence rate was high, and hence it was important to keep the patients on follow-up. It is mentioned in another study that there is currently no consensus regarding the frequency of follow-ups and the use of imaging techniques. Surveillance should be done every three to six months during the first three years following the diagnosis and every 6-12 months after that, depending upon the relapse-risk profile of the patient [21,22].

Half of the cases reported metastatic spread, which was most common in the lungs, followed by the bones and equally in the liver, brain, and lymph nodes. These stats can vary with change in the approach and study design. Local recurrence was high with surgery alone, and it tended to come down when the treatment involved palliative radiotherapy along with surgery. Mortality in our population was not significant as the survival rate was found to be 64.3% after one year. On follow-up, 18 out of 26 patients survived. While we lost four patients to follow-up, six patients deceased after undergoing the treatment.

We can conclude that CMM could be effectively treated with effective management, which includes implementing mass awareness programs and introducing screening tests for high-risk people. Identifying high-risk populations is a big challenge in our setup. Further studies should focus on the identification of high-risk people and the provision of inexpensive screening tools to control the rising trend. The data we collected is insufficient to form a definite opinion about the gender distribution of the disease and the correlation of several epidemiological factors. Based on our findings, surgical excision along with radiotherapy is the most effective treatment method.

## Conclusions

CMM is the third most common skin malignancy and the most fatal of all. Although the disease is commonly associated with fair-skinned populations, the incidence of CMM has been on the rise in our part of the world as well. Early diagnosis and prompt management of the disease are crucial for attaining favorable outcomes. However, the rates of recurrence and metastases remain high even when adequate management is provided. Due to the aggressive nature of the disease, the overall survival rate remains very low.
